# Continental Monophyly and Molecular Divergence of Peninsular Malaysia's *Macaca fascicularis fascicularis*

**DOI:** 10.1155/2014/897682

**Published:** 2014-07-17

**Authors:** Muhammad Abu Bakar Abdul-Latiff, Farhani Ruslin, Hamdan Faiq, Mohd Salleh Hairul, Jeffrine Japning Rovie-Ryan, Pazil Abdul-Patah, Salmah Yaakop, Badrul Munir Md-Zain

**Affiliations:** ^1^School of Environmental and Natural Resource Sciences, Faculty of Science and Technology Universiti Kebangsaan Malaysia, 43600 Bangi, Selangor, Malaysia; ^2^Department of Wildlife and National Parks (PERHILITAN), Km 10, Jalan Cheras, 50664 Kuala Lumpur, Malaysia

## Abstract

The phylogenetic relationships of long-tailed macaque (*Macaca fascicularis fascicularis*) populations distributed in Peninsular Malaysia in relation to other regions remain unknown. The aim of this study was to reveal the phylogeography and population genetics of Peninsular Malaysia's *M. f. fascicularis* based on the D-loop region of mitochondrial DNA. Sixty-five haplotypes were detected in all populations, with only Vietnam and Cambodia sharing four haplotypes. The minimum-spanning network projected a distant relationship between Peninsular Malaysian and insular populations. Genetic differentiation (*F*_ST_, Nst) results suggested that the gene flow among Peninsular Malaysian and the other populations is very low. Phylogenetic tree reconstructions indicated a monophyletic clade of Malaysia's population with continental populations (NJ = 97%, MP = 76%, and Bayesian = 1.00 posterior probabilities). The results demonstrate that Peninsular Malaysia's *M. f. fascicularis* belonged to Indochinese populations as opposed to the previously claimed Sundaic populations. *M. f. fascicularis* groups are estimated to have colonized Peninsular Malaysia ~0.47 million years ago (MYA) directly from Indochina through seaways, by means of natural sea rafting, or through terrestrial radiation during continental shelf emersion. Here, the Isthmus of Kra played a central part as biogeographical barriers that then separated it from the remaining continental populations.

## 1. Introduction

*Macaca fascicularis* [1], commonly known as the long-tailed macaque or crab-eating macaque, is called* kera* in Malaysia [2].* M. fascicularis* is probably the most successful nonhuman primate in Southeast Asia; long-tailed macaques are distributed in Malaysia, Brunei, Bangladesh, Cambodia, Nicobar Islands, Indonesia, Laos, Myanmar, the Philippines, Singapore, Thailand, Timor-Leste, and Vietnam [3]. In addition to its extensive geographical distribution, the opportunistic nature of* M. fascicularis* enables it to naturally inhabit a wide range of habitat, namely, primary and secondary forest, riverine, swamps, coastal areas, and mangrove forest from sea level up to an elevation of 2000 m [4, 5]. Furthermore, due to human introduction, this opportunistic species is able to survive in new habitats far from its natural distribution, specifically in the Pacific Ocean (Palau), Indian Ocean (Mauritius), and New Guinea [3].

**Figure 1:**
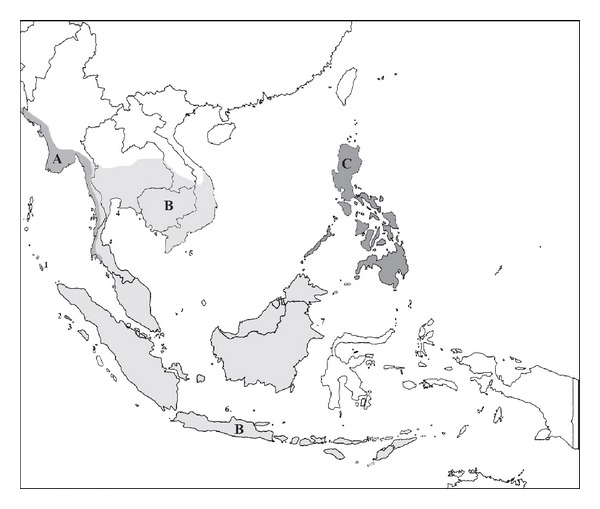
The distribution of 10 subspecies of* M. fascicularis*. A =* M. f. aurea*, B =* M. f. fascicularis*, C =* M. f philippinensis*. The isolated island subspecies are labeled with numbers. 1 =* M. f umbrosa*, 2 =* M. f. lasiae*, 3 =* M. f. fusca*, 4 =* M. f. atriceps*, 5 =* M. f. condorensis*, 6 =* M. f. karimondjawae*, and 7 =* M. f. tua* [3].

Long-tailed macaques have been classified into as many as 50 subspecies and even several different species [6], but the most recognized classifications are 10 subspecies of* M. fascicularis* [6–8]. The 10 subspecies of* M. fascicularis* that are presently recognized based on their morphological characteristics are as follows (Figure 1):* M. f. atriceps* (Kloss 1919),* M. f. aurea* (*Geoffroy 1831*),* M. f. condorensis* (Kloss 1926),* M. f. fascicularis* (Raffles 1821),* M. f. fusca* (Miller 1903),* M. f. karimondjawae* (Sody 1949),* M. f. lasiae* (Lyon 1916),* M. f. philippinensis* (*Geoffroy 1843*),* M. f. tua* (Kellog 1944), and* M. f. umbrosa* (Miller 1902) [6–8]. Only a single subspecies of the long-tailed macaque is distributed in Peninsular Malaysia, namely,* M. f. fascicularis* [1] and it is the most widely distributed compared to the other subspecies. In Peninsular Malaysia,* M. f. fascicularis* is found on the mainland peninsula and surrounding islands, such as Langkawi Island. Furthermore,* M. fascicularis* is at the center of human-wildlife conflict in this region, especially in secondary forest and palm oil plantations neighboring human settlements [2, 9].

Previous studies have extensively examined the molecular phylogeny and biogeography of* M. fascicularis* using various molecular data such as allozymes [10], mitochondrial DNA (mtDNA) [11–20], and nuclear data [11, 12, 14]. Blancher et al. [15] successfully defined the continental-insular populations of* M. fascicularis* in Southeast Asia, but whether Peninsular Malaysia's* M. fascicularis* belong to continental or insular groups remains unknown. Roos et al. [21] proposed that colobines may have invaded Eurasia, diversified into several lineages, and radiated from mainland Southeast Asia downwards to Java, Sunda, and Sumatra; therefore, it may be possible to suggest that Peninsular Malaysia's long-tailed macaque should be classified as continental. However, Peninsular Malaysia has been hypothesized to be the bridge that facilitated the radiation of nonhuman primates from Java to mainland Southeast Asia [22]. Thus, Peninsular Malaysia's* M. fascicularis* cannot simply be defined as a continental group as in [15], because it is found in mainland Southeast Asia.

In mammals, mtDNA is inherited as a haploid from the mother [23]. This gives a bigger picture of diversity in the particular gene pool of an organism caused by the occurrence and accumulation of mutations in mtDNA [24]. Moreover, mtDNA does not undergo DNA recombination [25]. All of these factors make mtDNA a very valuable tool when it comes to studying the relationships between populations. However, cases of mtDNA-derived nuclear pseudogenes (Numts) have been reported to arise in phylogenetic studies on primates [26]. The amplification of Numts rather than targeted mtDNA will disturb the molecular study of organisms due to the inclusion of paralogous nuclear sequences in the analysis instead of mtDNA sequences [27, 28]. This study will exploit the noncoding hypervariable region of mtDNA because this evolves more rapidly, thereby reflecting more differentiation among closely related taxa [29, 30].

Despite extensive studies on the molecular phylogeny of long-tailed macaques, the molecular data and phylogenetic position of Peninsular Malaysia's* M. fascicularis* in relation to other countries remain unknown. This may hold the key to revealing the biogeographic and radiation history of this species in Southeast Asia. Thus, the objectives of this study were to analyze the phylogeography and population genetics of Peninsular Malaysia's* M. f. fascicularis* through comparison with the available data on* M. f. fascicularis* from other countries using the D-loop region of mtDNA.

## 2. Methodology

### 2.1. DNA Extraction, Polymerase Chain Reaction (PCR), and Sequencing

**Figure 2:**
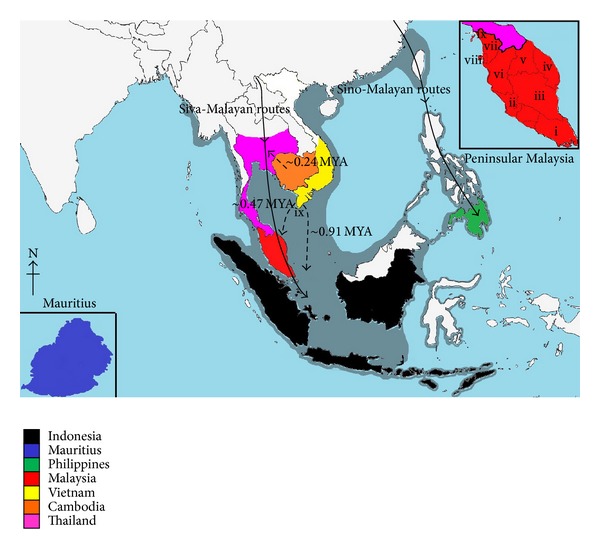
Map depicting the sampling location of* M. f. fascicularis* in Peninsula Malaysia (i-ALMFJ204,264,195; ii-FRMFB1-3; iii-ALMFC227-228; iv-ALMFT322; v-ALMFD16-17; vi-ALMFA63-64; vii-ALMFK109-112; viii-Penang samples from GenBank; ix-ALMFR370-371) and locality of sequence extracted from GenBank are color coded with respect to the legends provided. The gray shading represents continental shelf emersion [35]. Siva-Malayan and Sino-Malayan routes are also illustrated on the map.

A total of 23 fecal genetic samples of* M. f. fascicularis* were used in this study (Table 1). These were obtained with the assistance of the Department of Wildlife and National Parks Malaysia (PERHILITAN). All 23 samples were collected across mainland Peninsular Malaysia (Figure 2), while with the exception of Penang Island, no samples were used from surrounding islands, as there are some contradictions in the classifications of* M. f. fascicularis* [7, 8, 32–34]. The samples from Penang Island (10 sequences from GenBank) were confirmed as* M. f. fascicularis*. DNA was extracted from 0.5–1.0 g of fecal samples using the innuPREP Stool DNA Kit (Analytik Jena) following the manufacturer's protocol.

To conduct a comparative analysis of Malaysia's* M. f. fascicularis*, we used representatives of all* M. f. fascicularis* sequences available in GenBank for the mtDNA control region (CR). There were 253 available sequences. Two were from Java [16]; 77 from the Philippines, Indonesia, and Mauritius [15]; 9 from Java, Sumatra, Kalimantan, Bali, the Philippines, China, and Mauritius [17]; 10 from Thailand [18]; 45 from Penang, Malaysia [19]; and 95 from Cambodia, Vietnam, Indonesia, and the Philippines [20]. To avoid redundancy in the data analysis, 77 sequences of* M. f. fascicularis* were randomly selected from these 253 sequences (each representing a unique haplotype/clade with known locality information) across seven countries (Indonesia, Malaysia, Mauritius, the Philippines, Cambodia, Vietnam, and Thailand; Table 2). In addition, two sequences of* M. mulatta* and* M. sylvanus* were used as an outgroup to root the phylogenetic trees and as a calibration point for molecular clock rate estimation.

A 1,100 bp fragment of mtDNA D-loop was amplified through a polymerase chain reaction (PCR) using a Mastercycler Nexus (Eppendorf North America, Inc.). PCR reactions were generated using the Phusion Flash High-Fidelity PCR Master Mix (Finnzymes, OY), which has high accuracy (proofreading DNA polymerase with a fidelity of 25X* Taq polymerase*), extreme speed (extension times of 15 s/kb or less), and a very high yield in a reduced length of time. We designed our own primer for the PCR reactions in order to maximize the extent of the D-loop sequence and to avoid amplifying Numts. The primers used were LATIFF1638_F (5′-ACAGTCCTAGTATTAACCTGC-3′) and LATIFF1689_R (5′-CAAGGGGTGTTTAGTGAAGT-3′). The parameters for the PCR reaction were as follows: initial denaturation for 10 s at 98°C, followed by 30 cycles of denaturation for 1 s at 98°C, annealing for 30 s at 52°C, extension for 15 s at 72°C, and a final extension stage for 1 min at 72°C. Vivantis G-F1 PCR Clean-up Kits were used to purify the PCR product, and the samples were subsequently sent to 1st Base Sdn Bhd (Malaysia) for sequencing purposes.

### 2.2. Sequence and Phylogenetic Analysis

D-loop sequences obtained after the sequencing process were edited using Bioedit Sequence Alignment Editor. To ensure the targeted species locus sequences were obtained, the edited sequences were validated using sequence similarity searches (GenBank BLASTn). The MEGA 5 ClustalW multiple alignment algorithm was used to align all 102 sequences [36]. The aligned D-loop sequences were then analyzed at three different levels, namely, sequence analysis, phylogenetic analysis, and population genetics analysis. MEGA 5 [36] was heavily exploited in the sequence analysis, as well as PAUP 4.0b10 [37] and DnaSP 4.0 [38]. Sequence analyses are crucial in revealing a few key end results, such as genetic distance, single-nucleotide polymorphisms (SNPs), net nucleotide divergence (Da), and nucleotide diversity (*π*).

Population expansion events were inferred by employing mismatch distribution analysis [39, 40] using Arlequin ver. 3.1 with 1,000 permutations [41]. The haplotype relationships of* M. fascicularis* were reconstructed by assuming that at any given site, two randomly drawn haplotypes were unlikely to have arisen from more than one mutational step [42]. Network 4.6.1.2 was used to generate a minimum-spanning network (MSN). Genetic differentiation such as nucleotide subdivision (Nst) [43], population subdivision (*F*_ST_), and number of migrants per generation (*N*_*m*_) estimated using [44] were calculated in DnaSP 4.0.

The demographic history of* M. f. fascicularis* in Southeast Asia was examined by employing Tajima's test of neutrality, *D* [45], Fu and Li's D* and F* [46], and Fu's Fs [47]. Tajima's *D* test compares the average number of pairwise nucleotide differences (*k*) between haplotypes in a sample (M) expected from the number of segregating sites (*K*). Fu and Li's D* and F* and Fu's Fs were used to test for deviation of sequence variation from evolutionary neutrality. Fu's Fs is based on the probability of the observed number of haplotypes occurring under conditions of neutrality, whereas Fu and Li's D* and F* compare estimates of theta based on mutations in internal and external branches of a genealogy.

Phylogenetic trees were constructed using three distinct criteria, namely, distance-based (neighbor-joining (NJ) tree), character-based (maximum parsimony (MP) tree), and Bayesian inference. Different software programs were used to construct the phylogenetic tree, namely, MEGA 5 for the NJ tree [36], PAUP 4.0b10 for the MP tree [37], and MrBayes 3.1 for Bayesian inference [48]. The Kimura 2-parameter model was used in NJ tree reconstructions tested with a bootstrap value of 1,000, and the tree bisection and reconnection (TBR) algorithms were used for the MP tree. The heuristic searching method and 1,000 random stepwise additions were applied to find the best tree through the application of the 50% consensus majority rule. All the trees constructed underwent 1,000 bootstrap replications to obtain the bootstrap confidence level.

The best substitution model for D-loop sequences was selected using Modeltest version 3.7 software [49] by means of the Akaike information criterion (AIC) requirements. The best model for the sequences selected was the TrN+I+G with a gamma shape parameter of 0.5597 and base frequencies of 0.2915 for adenosine, 0.3310 for cytosine, 0.1035 for guanine, and 0.2741 for thymine. Metropolis-coupled Markov chain Monte Carlo (MCMC) was run with 10 million generations, and the tree was sampled every 1,000 generations. A split frequencies probability (*P*) of 0.006136 was obtained across two different runs of MrBayes. The first 10% of the trees obtained in the analysis were discarded as burn-in (1,000 trees discarded from a total of 10,000 trees), and a majority-rule consensus for the remaining trees was constructed; the posterior probabilities (*PP*) were summarized for each branch.

The divergence times of* M. fascicularis* in this study were estimated using BEAST version 1.7.5 [50]. Two datasets were defined in the analysis, namely, ingroup and outgroup, where the outgroup dataset included only* M. sylvanus*. The uncorrelated lognormal relaxed-clock model [51] was used to reconstruct the molecular divergence phylogenetic tree to estimate the substitution rate for all nodes in the tree with uniform priors on the mean (0,100) and standard deviation (0,10). The birth-death speciation model [52], which suggests that births and deaths of lineages occur at a constant rate and are independent, was used to reconstruct the starting tree, with the assumption that the ingroups were monophyletic with respect to the outgroup.* M. sylvanus* was used to root the ingroups and as the most recent common ancestor (TMRCA) or calibration point, estimated around 5.5 million years ago (MYA) based on fossil data [53, 54]. MCMC was run for 10 million generations and the trees were sampled every 1,000 generations, with 1% of the sample discarded as burn-in. Tracer version 1.5 was used to assess the estimated sample size (ESS) from the log files produced by BEAST. After 10 million generations, the ESS of all parameters (posterior, prior, likelihood, ucld.mean, etc.) well exceeded 200, suggesting that the MCMC steps were more than adequate. The maximum-clade-credibility tree topologies were calculated using posterior distribution, and TreeAnnotator version 1.7.5 was employed to produce the final summary trees; finally, FigTree version 1.4.0 was used to view the tree.

## 3. Results

D-loop sequences as low as 1,000 bp were successfully sequenced for all 23 genetic samples used in this study (Table 1). All samples matched the same GenBank sequences, JX113341, from Penang with a minimum score of 95% query cover when blasted for sequence similarity searches using GenBank BLASTn. Although we successfully sequenced more than 1,000 bp of the D-loop fragment, when we compared it with the sequence available on GenBank for comparative analysis purposes, most of the D-loop sequences were HVSII [15]. Consequently, we aligned our sequence with the sequence available from GenBank (Table 2) and only used the HVSII region (~400 bp) for this study.

A total of 395 bp D-loop sequences of the HVSII region were obtained for sequence, phylogenetic, and population genetic analyses. It was found that 291 (73.6%) out of 395 characters in the sequences were constant, leaving 104 (26.3%) variable characters. Eighty one (20.5%) characters were parsimony informative, while the 23 (5.8%) remaining characters were parsimony uninformative (Table 3). The sequence showed an average of 28.1% of thymine, 32.3% of cytosine, 28.4% of adenosine, and 11.2% of guanine in the sequence across all taxa.

Average pairwise distances among* M. f. fascicularis* (population as in the country they are distributed in) based on the Kimura 2-parameter model were also calculated (Table 4). The pairwise genetic distance exhibits a model of the relationship between the populations of* M. f. fascicularis*, namely, the continental populations (Thailand, Cambodia, Vietnam, and Malaysia) and the insular populations (Indonesia, Philippines, and Mauritius). The genetic distance between these two groups (continental-insular) was relatively high compared to distance within the groups. For instance, the genetic distance between Malaysia and Vietnam was only 0.033, but with the three insular populations (Mauritius, Indonesia, and Philippines), it was as high as 0.098. In contrast, the distances within the groups were only 0.026–0.050 (continental) and 0.026–0.031 (insular). SNP analysis conducted on the D-loop sequences revealed 104 SNPs throughout the sequences analyzed.

Nucleotide diversity (*π*) and net nucleotide divergence (Da) among* M. fascicularis* populations revealed the same model of continental-insular relationships and separate Peninsular Malaysian populations from insular populations (Table 5). Within the insular group, only a maximum value of 0.02860 (Indonesia-Mauritius) for *π* and 0.02025 (Mauritius-Philippines) for Da was obtained, similar to the continental group, with 0.03227 for *π* (Malaysia-Vietnam) and 0.03163 for Da (Malaysia-Thailand). However, the analysis between the continental and insular groups showed a relatively high value for both *π* and Da, a minimum value of 0.03212 (Malaysia-Mauritius) and 0.04562 (Indonesia-Vietnam), respectively.* M. f. fascicularis* populations in Peninsular Malaysia, Thailand, Cambodia, and Vietnam were included in the continental group. The Philippines, Mauritius, and Indonesia were included in the insular group for additional analysis and were found to exhibit 0.05101 for *π* values and 0.04549 for Da.

Genetic differentiation (*F*_ST_, Nst, *N*_*m*_) values were calculated to further elucidate the relationships among* M. f. fascicularis* populations in seven different countries (Table 5). *F*_ST_ is the probability that two random gametes drawn from two populations are identical by descent and relative to gametes taken from the whole populations. Only *F*_ST_ values >0.25 strongly indicate a genetic differentiation of populations [55].* M. f. fascicularis* from all five countries showed a significant subdivision from one another, with a minimum value of *F*_ST_ of 0.307 (Indonesia-Philippines), except for Cambodia-Vietnam (0.001). The lowest *F*_ST_ value between continental-insular groups was 0.605 (Indonesia-Vietnam), in contrast to Mauritius populations, which showed the highest division from Thailand, with 0.992. Nst analysis can be used to estimate a population's subdivision at the nucleotide level [56], with 0 = no population subdivision and 1 = complete population division. Nst analysis outcomes were completely parallel with *F*_ST_, with Cambodia-Vietnam exhibiting lowest Nst of 0.00, followed by Philippines-Indonesia populations with 0.31. The division of Mauritius populations from Thailand revealed the highest Nst, at 0.99. Theoretically, when the *N*_*m*_ value is <1, populations are expected to genetically diverge over time, and when *N*_*m*_ is >1, they are expected to retain gene flow. Thus, the *N*_*m*_ value will be inversely proportional to both *F*_ST_ and Nst. Here, *N*_*m*_ analysis validated both *F*_ST_ and Nst, as Cambodia-Vietnam had the highest *N*_*m*_, with an astonishing value of 494.53 with the same population size (15 for each population). *N*_*m*_ describes the average number of individuals per generation migrating between populations; thus, there appear to be nearly 500 individuals migrating between Cambodia and Vietnam per generation, retaining their gene flow as a result. The *N*_*m*_ value for Mauritius-Thailand was the lowest, at 0.00, suggesting that the gene flow between these two populations was cut off over time.

**Figure 3:**
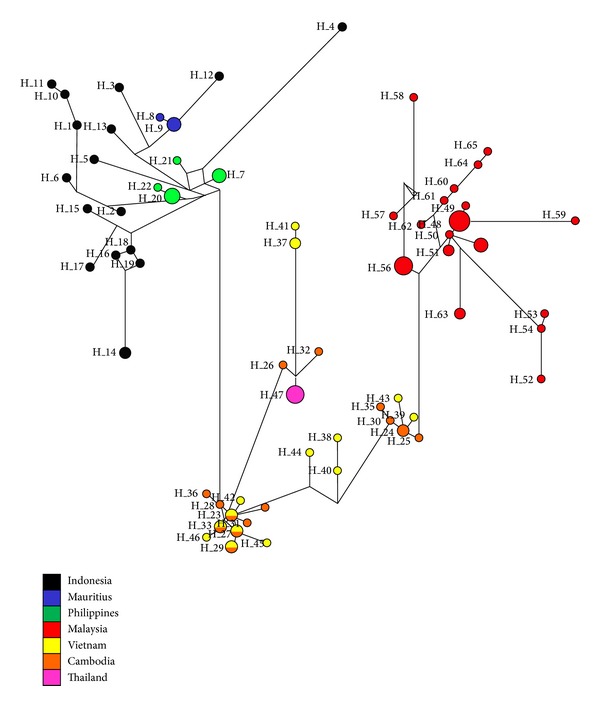
The minimum-spanning network (MSN) generated by Network 4.6.1.2 [57] illustrating the relationships of the long-tailed macaques,* M. f. fascicularis* in seven countries. Each circle represents a haplotype, and the diameter is scaled to the haplotype frequency.

Sixty-five haplotypes with a size of 104 bp were defined from the seven populations of* M. f. fascicularis* (Table 6) and obtained from 100 analyzed sequences (excluding outgroups). Populations originating from Malaysia, Indonesia, Cambodia, and Vietnam exhibited high haplotype diversity (Hd > 0.9), with Mauritius populations having considerably lower haplotype diversity (Hd = 0.500 ± 0.265); this coincides with the *π* of the haplotype, with Mauritius having the lowest value of 0.00127 ± 0.00067. Cambodia and Vietnam are the only populations that share the same haplotypes (Hap_23, Hap_27, Hap_29, and Hap_33; Table 7). Thailand populations only contain one unique haplotype, Hap_47; Malaysian populations, on the other hand, have the highest number, with 18 unique haplotypes (Hap_48–Hap_65). MSN was generated with the haplotype data obtained to illustrate the relationships of the seven populations of* M. f. fascicularis* (Figure 3). The network analysis revealed that Peninsular Malaysia's* M. f. fascicularis* is more related to continental populations (Vietnam, Cambodia, and Thailand), as there are far fewer mutational steps between the populations as compared to insular populations. The analysis also portrayed Cambodia/Vietnam as the connecting point for both populations from Peninsular Malaysia and insular populations; the fewest mutational steps to their respective haplotypes were found.

**Figure 4:**
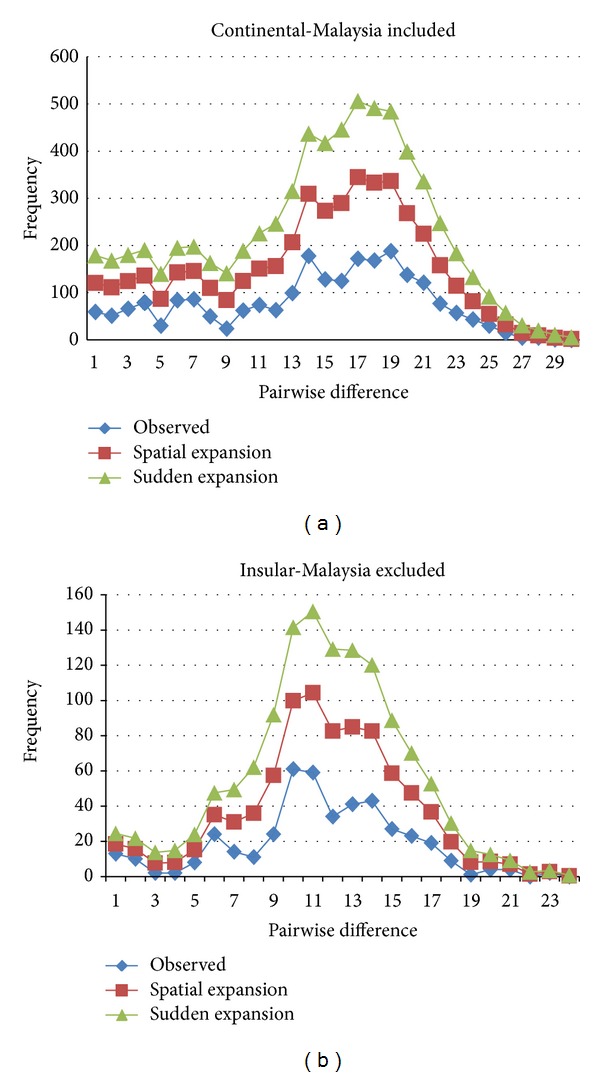
Mismatch distribution of expected and observed frequencies of pairwise differences among HVSII sequences of continental and insular groups of* M. f. fascicularis* under the sudden and spatial expansion models.

Mismatch distribution of pairwise nucleotide differences of the HVSII sequences was estimated to study the demographic history of* M. fascicularis* populations in Southeast Asia using the seven populations in this study as a model. By assuming that the amount of difference between alleles depends directly on the length of time since they diverged, we could manipulate the whole distribution of sequence differences to observe the demographic expansion of* M. fascicularis*. Thus, mismatch distributions of continental (Malaysia included) and insular (Malaysia excluded) groups (Figure 4) were carried out following the expected distribution under a sudden expansion model [40, 58] and a spatial expansion model [41, 59] to portray these events. The continental group's mismatch distribution exhibited a multimodal expansion pattern by means of the sum of squared deviation (SSD) = 0.0023, with significance observed of *P* = 0.88 and Harpending's raggedness index = 0.0054 with significance observed at *P* = 0.76. The insular groups also indicated a multimodal expansion distribution by means of SSD = 0.074 with *P* = 0.26 and Harpending's raggedness index = 0.0168 with *P* = 0.14. The mismatch distribution of pairwise nucleotide differences among HVSII sequences for both continental and insular populations exhibited ragged multimodal distribution characteristics of population expansion and revealed that the observed distribution is parallel with the expected distribution under the sudden (recent) expansion model [40, 58] and the spatial expansion model (range expansion with high levels of migration between neighboring demes [41, 59]). The multimodal expansion pattern usually reflects a highly stochastic shape of gene pool or a recent demographic expansion.

**Figure 5:**
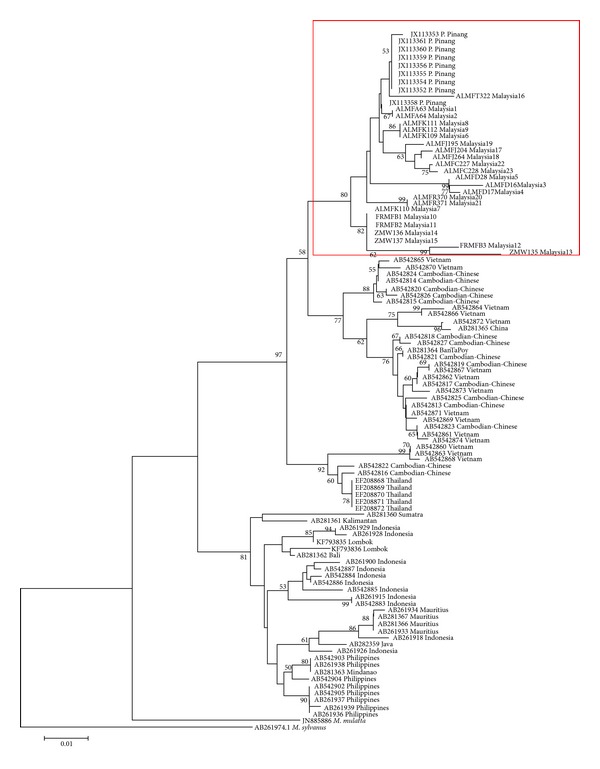
The neighbor-joining phylogenetic tree estimated using the Kimura-2-parameter algorithm and 1000 bootstrap replications. The optimal tree with the sum of branch length = 0.6544 is shown and bootstrap values are indicated on the branches.

**Figure 6:**
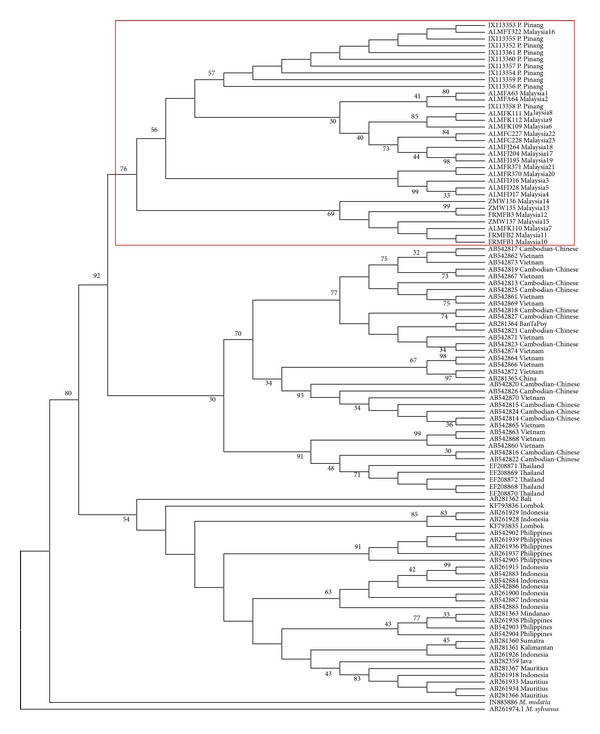
The maximum parsimony (MP) phylogenetic tree estimated using the TBR algorithm, heuristic searching method, and 1000 bootstrap replications. Bootstrap values are shown on the branches. (Tree length = 264, CI = 0.4009, RI = 0.8944, HI = 0.4337).

**Figure 7:**
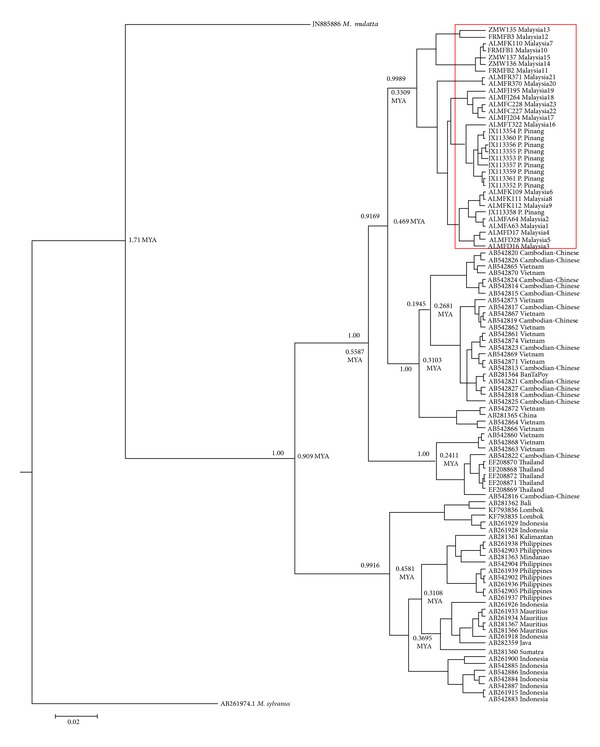
Bayesian inference of the 50% majority rule consensus and the molecular divergence tree of the HVSII sequence of* M. f. fascicularis* populations with Bayesian posterior probability (*PP*) are accordingly indicated on the branches. The numbers on the nodes represent divergence time in millions of years (MYA).

The reconstructed NJ phylogenetic tree (Figure 5) showed a monophyletic formation of ingroup samples (*M. f. fascicularis*), supported with a 79% bootstrap value. Two clades of* M. fascicularis* populations were further defined, namely, the continental groups (Peninsular Malaysia, Cambodia, Thailand, and Vietnam) and insular groups (the Philippines, Indonesia, and Mauritius), supported by 97% and 81% bootstrap values, respectively. The MP phylogenetic tree (Figure 6) and Bayesian inference phylogenetic tree (Figure 7) likewise supported the hypothesis that Peninsular Malaysia's populations are continental populations and revealed continental-insular clade formation, supported by a 92% bootstrap value/1.00 posterior probabilities and a 54% bootstrap value/0.9916 posterior probabilities, respectively. The molecular divergence phylogenetic tree (Figure 7) was constructed using the uncorrelated lognormal relaxed-clock model. This was done to estimate the substitution rate for all nodes in the tree to establish the divergence dates of Peninsular Malaysia's* M. f. fascicularis* populations as compared to other countries' populations. By exploiting* M. sylvanus* samples as a calibration point and TMRCA, the analysis indicated that* M. f. fascicularis* groups diverged from* M. mulatta* ~1.71 MYA. Continental-insular populations diverged at ~0.91 MYA, followed by another divergence of continental populations at ~0.47 MYA that separated populations of* M. f. fascicularis* in Peninsular Malaysia from the rest of continental Southeast Asia.

## 4. Discussion

The analysis in this study has shown that the* M. fascicularis* subspecies group forms a monophyletic clade as compared to* M. mulatta* and despite reported cases of introgression of* M. mulatta* and* M. fascicularis* based on Y-chromosome investigation [11]. In Peninsular Malaysia,* M. fascicularis* exists sympatrically with* M. nemestrina*; thus, a case of introgression is a possibility, but by employing mtDNA as molecular mtDNA, we could infer the phylogenetic relationships of targeted groups more precisely. This represents the first study to analyze the comparative phylogenetic position of* M. f. fascicularis* in Peninsular Malaysia, although it is the centerpiece of the connection between insular and continental populations of* M. fascicularis* in Southeast Asia. The hypervariable locus of mtDNA has proven to be effective in resolving the phylogeny of* M. f. fascicularis* populations in Southeast Asia, consistent with Blancher et al.'s work [15].

Geographical (distance and barriers) and anthropological (deforestation, land conversion, and habitat destruction) factors may play a crucial role in separation of* M. f. fascicularis* populations in Southeast Asia. The mismatch distribution analysis revealed a multimodal expansion pattern, which heavily suggests a recent demographic expansion, parallel with the opportunistic nature of the long-tailed macaque. Instead of being threatened due to an inability to survive in the face of habitat destruction,* M. fascicularis* group adapt to inhabit areas neighboring human settlements where they will have access to gardens, farms, and even houses to crop raid [60, 61].

### 4.1. The Dispersal Mechanism of* M. f. fascicularis fascicularis* in Southeast Asia

Population genetic analysis of Peninsular Malaysia's* M. fascicularis* revealed a complete separation of the populations from six populations in other countries. While it showed a closer relationship to populations originating from Vietnam, Cambodia, and Thailand as compared to other insular populations, the results (*F*_ST_, Nst, and *N*_*m*_) suggest an almost cut-off gene flow concerning the rest of the continental populations. Not a single haplotype is shared between the Peninsular Malaysian population and the rest of the continental populations, and MSN analysis clearly showed the distant relationship between all the populations, particularly continental-insular groups. Furthermore, the network analysis (Figure 3) revealed a more geographic relationship of* M. f. fascicularis *populations in Southeast Asia, as the insular populations were more closely related to those in Cambodia and Vietnam. This is unprecedented, as Peninsular Malaysia has been hypothesized to act as a connecting bridge for the Sundaic populations, allowing radiation of primates between mainland Southeast Asia and its insular area [21, 22]; thus, it should have much more closer relationships with the insular populations.

Our hypothesis is that the radiation of* M. f. fascicularis* possibly began from the Indochinese region and underwent two different dispersal mechanisms that led to the formation of insular lineages and the colonization of the Malay Peninsula (Figure 2). First, the long-tailed macaque radiated from Indochina to the Sunda shelf around ~0.91 MYA; this population subsequently diverged and colonized a different part of the Sunda shelf. Second,* M. f. fascicularis* radiated to Peninsular Malaysia ~0.47 MYA directly from Indochina via seaways, colonized the area, and remained separated from the rest of the continental populations. The Isthmus of Kra played a pivotal role as a barrier to gene flow. These suggestions are congruent with the fossil records collected in the Sunda shelf as early as at least the later Early Pleistocene around ~1 MYA, from Trinil, east central Java [57]. During the glacial periods of the Pleistocene, the fluctuations of sea level led to the emersion of a huge continental shelf extending to marine areas estimated at around 200 m in depth (Figure 2) [35]. If this is the case, then the radiation of* M. f. fascicularis* by land is possible.

Alternatively, in the event that the emersion of the continental shelf did not overlap during the radiation period of* M. f. fascicularis*, sea level fluctuations before 0.8 MYA were moderate, with a mean of 70 m and the lowest sea levels at around 100 m below that of the present day, which may have also facilitated the radiation [62]. One of the prime habitats of the widely adaptive* M. f. fascicularis* is coastal habitat; long-tailed macaques are excellent swimmers, even underwater [6], making the passive dispersal theory of natural sea rafting by means of the Siva-Malayan route a fitting idea for the dispersal of the species.

Minimum-spanning network analysis (Figure 3), genetic distances, and population genetic analysis revealed that populations from the Philippines are much more closely related to Indochinese populations. If by any chance* M. f. fascicularis* dispersed to the Philippines via the Siva-Malayan route, then it should have a closer relationship to Peninsular Malaysia rather than to Indochinese populations. Based on Chinese affinities of the fauna found in the Philippines but not on the Sunda shelf, the authors in [63, 64] proposed an alternate migration route of mammals during the Pleistocene in which they reached Java via the Philippines and Borneo. This theory might explain the closer genetic distance of* M. f. fascicularis* in the Philippines to Indochinese populations as compared to Peninsular Malaysia's. However, this theory has largely been discredited [65], as Sulawesi shared similar island faunas, suggesting that they were not part of a continuous land migration route. Alternatively, the haplotypes observed might possibly be derived from a single ancestral haplotype with low genetic and nucleotide diversities within the Philippine populations [15].

Indeed, the dispersal theory in the field of biogeography relies heavily on fossil and geological records, which comprise the main pillar of the theory. For example, in the case of the radiation history of* Pongo pygmaeus*, fossil remains from the Late Middle Pleistocene were found in South China, Vietnam, Laos, Cambodia, and Thailand, and the same fossils were discovered in Late Pleistocene sites in Indonesia [65]. A sea level fall ~70,000 years ago, after the glacial maximum ~135,000 years ago, might have initiated the migration between the mainland and Indonesia and Borneo [66]. However, it is almost impossible to illustrate the migration routes of the other Indochinese species, whether they are limited southward to Thailand or Peninsular Malaysia, because of the near total lack of Pleistocene fossiliferous sites in Peninsular Malaysia. The fossil discovery of extinct species of* Equus namadicus* with Indochinese characteristics in Tambun, Peninsular Malaysia [57], makes clear that it is possible that Peninsular Malaysia is not a definite Sundaic region. This supports the findings of this study.

### 4.2. The Role of the Isthmus of Kra in the Colonization of Peninsular Malaysia by* M. f. fascicularis*

The oriental biogeographical regions that comprise the Indochinese, Sundaic, and Wallacean provinces are an important part of the mysterious history of faunal dispersal and radiation in Southeast Asia [67]. The boundary between the Indochinese and Sundaic regions is claimed to be located at the Isthmus of Kra in Peninsular Thailand [67], as distinct assemblages of mammals [68] have been observed between the two ends of this barrier. However, reviews based on fossil records by [65] on the Pleistocene distribution of large mammals (129 extant species of large mammals, including primates) concluded that the biogeographical barriers to northern Peninsular Malaysia lay much farther south of the Isthmus of Kra during Pleistocene. Therefore, the southern biogeographical transitions on Peninsular Malaysia/Thailand lie approximately 500 km south of the Isthmus of Kra near the Thailand-Malaysia border [69].

This southern transition also involves a distinctive change between perhumid evergreen rainforest and wet seasonal evergreen rainforest [70–72] but has not necessarily affected the long-tailed macaques' radiation and distribution, as there is no adequate evidence that they are influenced by climate and environmental changes [73]. In the case of* M. f. fascicularis*, they are classified as a northern-southern group (with respect to the Isthmus of Kra) based on morphological and genetic traits [6, 10–12, 74].

Tosi and Coke [14] proposed the Isthmus of Kra as a biogeographical barrier to the monophyletic clade of* M. f. fascicularis* populations that does not undergo introgression with* M. mulatta* because the Y-chromosome of* M. mulatta* detected in* M. fascicularis* north of the Isthmus of Kra is absent in the southern populations. The differences discovered in fossil records, the variety of vegetation, and the genetic and morphological traits of* M. f. fascicularis* have led to the assumption that the differences have arisen because they belong to distinct biogeographical regions. The southern groups are always assumed to be the Sundaic population, and these deviate from the characteristics of those in Indochinese region. The phylogenetic analysis (NJ, MP, and Bayesian inference) in this study indicated a definite separation of Peninsular Malaysia's* M. f. fascicularis* from insular populations and the monophyletic state of Peninsular Malaysia's population, which is more closely related to Indochinese populations.

The estimation of divergence time of* M. f. fascicularis* populations in Malaysia indicates that the colonization in Peninsular Malaysia occurred roughly ~0.47 MYA, which is largely congruent with Tosi and Coke's [14] estimation of ~0.44 MYA based on Y-chromosomal DNA and Blancher et al.'s [15] study, which dated the separations of continental-insular populations at ~0.55 MYA. The same biogeographical barriers may have played a vital role in facilitating the colonization process of Peninsular Malaysia's* M. f. fascicularis* after the radiation of the species from Indochinese regions via terrestrial radiation during continental shelf emersion or natural sea rafting.

The biogeographical history of Southeast Asia is complex due to active tectonic plate movement, the rise and fall of sea level during the Pliocene and Pleistocene, drastic climate change, and the temporary formation of land bridges connecting mainland Southeast Asia to Sunda, Java, Sumatra, and Borneo [75–78]. These elements ultimately make it difficult to predict the dispersal patterns of* M. fascicularis* in Southeast Asia.

## 5. Conclusion

Populations of* M. f. fascicularis* in Peninsular Malaysia were found to be monophyletic in all phylogenetic analyses, with the absence of shared haplotypes with the other populations in Southeast Asia. This demonstrates that the species should be treated as a single unique lineage of long-tailed macaques in Southeast Asia. Nevertheless, populations from Malaysia are much more closely related to populations in Indochinese regions compared to insular populations, thus supporting the view that Peninsular Malaysia's populations are continental populations belonging to the Indochinese biogeographical regions, as opposed to Sundaic populations. The results of this study are crucial in the field of biogeography, as in the case of* M. f. fascicularis*; the inclusion of Peninsular Malaysia in the Sundaic regions is unsuitable.

## Figures and Tables

**Table 1:** Details on the samples used in this study.

No.	Sample name	Taxon	Locality
(1)	ALMFA63	*M. f. fascicularis *	Malay Peninsula
(2)	ALMFA64	*M. f. fascicularis *	Malay Peninsula
(3)	ALMFD16	*M. f. fascicularis *	Malay Peninsula
(4)	ALMFD17	*M. f. fascicularis *	Malay Peninsula
(5)	ALMFD28	*M. f. fascicularis *	Malay Peninsula
(6)	ALMFK109	*M. f. fascicularis *	Malay Peninsula
(7)	ALMFK110	*M. f. fascicularis *	Malay Peninsula
(8)	ALMFK111	*M. f. fascicularis *	Malay Peninsula
(9)	ALMFK112	*M. f. fascicularis *	Malay Peninsula
(10)	FRMFB1	*M. f. fascicularis *	Malay Peninsula
(11)	FRMFB2	*M. f. fascicularis *	Malay Peninsula
(12)	FRMFB3	*M. f. fascicularis *	Malay Peninsula
(13)	ZMW135	*M. f. fascicularis *	Malay Peninsula
(14)	ZMW136	*M. f. fascicularis *	Malay Peninsula
(15)	ZMW137	*M. f. fascicularis *	Malay Peninsula
(16)	ALMFT322	*M. f. fascicularis *	Malay Peninsula
(17)	ALMFJ204	*M. f. fascicularis *	Malay Peninsula
(18)	ALMFJ264	*M. f. fascicularis *	Malay Peninsula
(19)	ALMFJ195	*M. f. fascicularis *	Malay Peninsula
(20)	ALMFR370	*M. f. fascicularis *	Malay Peninsula
(21)	ALMFR371	*M. f. fascicularis *	Malay Peninsula
(22)	ALMFC227	*M. f. fascicularis *	Malay Peninsula
(23)	ALMFC228	*M. f. fascicularis *	Malay Peninsula

**Table 2:** The HVSII region of* M. f. fascicularis* sequences obtained from GenBank.

No.	Sample name	Taxon	Locality
(1)	KF793835^a^	*M. f. fascicularis *	Lombok Island
(2)	KF793836^a^	*M. f. fascicularis *	Lombok Island
(3)	AB281359^b^	*M. f. fascicularis *	Jatibarang, Java
(4)	AB281360^b^	*M. f. fascicularis *	Tabuan, Sumatra
(5)	AB281361^b^	*M. f. fascicularis *	Pangkalanbun, Kalimantan
(6)	AB281362^b^	*M. f. fascicularis *	Ubud, Bali, Indonesia
(7)	AB281363^b^	*M. f. fascicularis *	Cotabato, Mindanao, Philippines
(8)	AB281364^b^	*M. f. fascicularis *	Ban Tapoy, Laos
(9)	AB281365^b^	*M. f. fascicularis *	China
(10)	AB281366^b^	*M. f. fascicularis *	Mauritius
(11)	AB281367^b^	*M. f. fascicularis *	Mauritius
(12)	EF208868^c^	*M. f. fascicularis *	Khao Nor, Thailand
(13)	EF208869^c^	*M. f. fascicularis *	Khao Nor, Thailand
(14)	EF208870^c^	*M. f. fascicularis *	Khao Nor, Thailand
(15)	EF208871^c^	*M. f. fascicularis *	Khao Nor, Thailand
(16)	EF208872^c^	*M. f. fascicularis *	Thailand
(17)	JX113352^d^	*M. f. fascicularis *	Pulau Pinang, Malaysia
(18)	JX113353^d^	*M. f. fascicularis *	Pulau Pinang, Malaysia
(19)	JX113354^d^	*M. f. fascicularis *	Pulau Pinang, Malaysia
(20)	JX113355^d^	*M. f. fascicularis *	Pulau Pinang, Malaysia
(21)	JX113356^d^	*M. f. fascicularis *	Pulau Pinang, Malaysia
(22)	JX113357^d^	*M. f. fascicularis *	Pulau Pinang, Malaysia
(23)	JX113358^d^	*M. f. fascicularis *	Pulau Pinang, Malaysia
(24)	JX113359^d^	*M. f. fascicularis *	Pulau Pinang, Malaysia
(25)	JX113360^d^	*M. f. fascicularis *	Pulau Pinang, Malaysia
(26)	JX113361^d^	*M. f. fascicularis *	Pulau Pinang, Malaysia
(27)	AB261938^e^	*M. f. fascicularis *	Philippines
(28)	AB261939^e^	*M. f. fascicularis *	Philippines
(29)	AB261937^e^	*M. f. fascicularis *	Philippines
(30)	AB261936^e^	*M. f. fascicularis *	Philippines
(31)	AB261929^e^	*M. f. fascicularis *	Indonesia
(32)	AB261928^e^	*M. f. fascicularis *	Indonesia
(33)	AB261934^e^	*M. f. fascicularis *	Mauritius
(34)	AB261933^e^	*M. f. fascicularis *	Mauritius
(35)	AB261918^e^	*M. f. fascicularis *	Indonesia
(36)	AB261926^e^	*M. f. fascicularis *	Indonesia
(37)	AB261915^e^	*M. f. fascicularis *	Indonesia
(38)	AB261900^e^	*M. f. fascicularis *	Indonesia
(39)	AB542813^f^	*M. f. fascicularis *	Cambodian-Chinese
(40)	AB542814^f^	*M. f. fascicularis *	Cambodian-Chinese
(41)	AB542815^f^	*M. f. fascicularis *	Cambodian-Chinese
(42)	AB542816^f^	*M. f. fascicularis *	Cambodian-Chinese
(43)	AB542817^f^	*M. f. fascicularis *	Cambodian-Chinese
(44)	AB542818^f^	*M. f. fascicularis *	Cambodian-Chinese
(45)	AB542819^f^	*M. f. fascicularis *	Cambodian-Chinese
(46)	AB542820^f^	*M. f. fascicularis *	Cambodian-Chinese
(47)	AB542821^f^	*M. f. fascicularis *	Cambodian-Chinese
(48)	AB542822^f^	*M. f. fascicularis *	Cambodian-Chinese
(49)	AB542823^f^	*M. f. fascicularis *	Cambodian-Chinese
(50)	AB542824^f^	*M. f. fascicularis *	Cambodian-Chinese
(51)	AB542825^f^	*M. f. fascicularis *	Cambodian-Chinese
(52)	AB542826^f^	*M. f. fascicularis *	Cambodian-Chinese
(53)	AB542827^f^	*M. f. fascicularis *	Cambodian-Chinese
(54)	AB542860^f^	*M. f. fascicularis *	Vietnam
(55)	AB542861^f^	*M. f. fascicularis *	Vietnam
(56)	AB542862^f^	*M. f. fascicularis *	Vietnam
(57)	AB542863^f^	*M. f. fascicularis *	Vietnam
(58)	AB542864^f^	*M. f. fascicularis *	Vietnam
(59)	AB542865^f^	*M. f. fascicularis *	Vietnam
(60)	AB542866^f^	*M. f. fascicularis *	Vietnam
(61)	AB542867^f^	*M. f. fascicularis *	Vietnam
(62)	AB542868^f^	*M. f. fascicularis *	Vietnam
(63)	AB542869^f^	*M. f. fascicularis *	Vietnam
(64)	AB542870^f^	*M. f. fascicularis *	Vietnam
(65)	AB542871^f^	*M. f. fascicularis *	Vietnam
(66)	AB542872^f^	*M. f. fascicularis *	Vietnam
(67)	AB542873^f^	*M. f. fascicularis *	Vietnam
(68)	AB542874^f^	*M. f. fascicularis *	Vietnam
(69)	AB542883^f^	*M. f. fascicularis *	Indonesia
(70)	AB542884^f^	*M. f. fascicularis *	Indonesia
(71)	AB542885^f^	*M. f. fascicularis *	Indonesia
(72)	AB542886^f^	*M. f. fascicularis *	Indonesia
(73)	AB542887^f^	*M. f. fascicularis *	Indonesia
(74)	AB542902^f^	*M. f. fascicularis *	Philippines
(75)	AB542903^f^	*M. f. fascicularis *	Philippines
(76)	AB542904^f^	*M. f. fascicularis *	Philippines
(77)	AB542905^f^	*M. f. fascicularis *	Philippines
(78)	JN885886^g^	*M. mulatta *	
(79)	AB261974^e^	*M. sylvanus *	

^a^Wandia et al. [16]; ^b^Kawamoto et al. [17]; ^c^Malaivijitnond et al. [18]; ^d^Rovie-Ryan et al. [19]; ^e^Blancher et al. [15]; ^f^Shiina et al. [20]; ^g^Yu et al. [31].

**Table 3:** Summary of sequences analyzed across seven populations of *M. f. fascicularis*.

Total characters examined	395
Constant characters	291
Parsimony-uninformative characters	23
Parsimony-informative characters	81
% informative no. characters	20.5%
Ratio of TI/TV calculated from pairwise base differences	10.1

**Table 4:** Average pairwise distances among *M. f. fascicularis *populations based on the Kimura-2-parameter model.

	1	2	3	4	5	6
Indonesia						
Philippines	0.028					
Mauritius	0.031	0.026				
Cambodia	0.077	0.068	0.086			
Vietnam	0.081	0.073	0.089	0.026		
Thailand	0.074	0.067	0.086	0.033	0.036	
Malaysia	0.086	0.080	0.098	0.044	0.050	0.042

**Table 5:** Measures of nucleotide diversity (*π*), net nucleotide divergence (Da), nucleotide subdivision (Nst), estimate of population subdivision (*F*_ST_), and gene flow (number of migrants, *N*_*m*_) among populations of *M. f. fascicularis*.

Populations	*π*	Da	Nst	*F*_ST_	*N*_*m*_
Malaysia-Indonesia	0.04757	0.05621	0.71452	0.70427	0.21
Malaysia-Mauritius	0.03212	0.08012	0.89728	0.89241	0.06
Malaysia-Philippines	0.03696	0.06079	0.82756	0.82108	0.11
Malaysia-Cambodia	0.02907	0.02341	0.55392	0.54877	0.41
Malaysia-Vietnam	0.03227	0.02358	0.49767	0.49315	0.51
Malaysia-Thailand	0.02310	0.03163	0.78033	0.77801	0.14
Indonesia-Mauritius	0.02860	0.01469	0.49134	0.49128	0.52
Indonesia-Philippines	0.02589	0.00831	0.30647	0.30655	0.62
Indonesia-Cambodia	0.04930	0.04713	0.66516	0.65523	0.26
Indonesia-Vietnam	0.05321	0.04562	0.61571	0.60498	0.33
Indonesia-Thailand	0.04270	0.05482	0.79529	0.78997	0.13
Mauritius-Philippines	0.01558	0.02025	0.80855	0.80672	0.12
Mauritius-Cambodia	0.04039	0.06834	0.86798	0.86293	0.08
Mauritius-Vietnam	0.04752	0.06622	0.81271	0.80690	0.12
Mauritius-Thailand	0.04416	0.07848	0.99242	0.99200	0.00
Philippines-Cambodia	0.04002	0.04922	0.77953	0.77315	0.15
Philippines-Vietnam	0.04597	0.04864	0.72125	0.71452	0.20
Philippines-Thailand	0.03450	0.05879	0.93535	0.93304	0.04
Cambodia-Vietnam	0.02545	0.00003	0.00004	0.00101	494.53
Cambodia-Thailand	0.02382	0.02151	0.67936	0.67781	0.24
Vietnam-Thailand	0.03067	0.01989	0.56507	0.56662	0.38
Continental-Insular	0.05101	0.04549	0.61388	0.60272	0.33

**Table 6:** Summary statistic of D-loop mtDNA sequence variations in seven populations of *M. f. fascicularis*.

Populations	*N*	*H*	*S*	Hd^†^	*π* ^†^	*K*	*D*	Fs	D*	F*
Malaysia	33	18	45	0.9191 ± 0.032	0.01805 ± 0.00264	7.131	−1.30238	−3.422	−1.05396	−1.34515
Indonesia	17	16	44	0.993 ± 0.023	0.02915 ± 0.00271	11.515	−0.47866	−5.846	−0.62676	−0.67653
Mauritius	4	2	1	0.500 ± 0.265	0.00127 ± 0.00067	0.500	−0.61237	0.172	−0.61237	−0.47871
Philippines	9	4	8	0.750 ± 0.01257	0.00844 ± 0.00143	3.333	0.60112	1.546	0.13479	0.27529
Cambodian-Chinese	15	14	27	0.990 ± 0.028	0.02045 ± 0.00294	8.076	−0.11460	−6.054	−0.01858	−0.05199
Vietnam	15	14	40	0.990 ± 0.028	0.03043 ± 0.00436	12.019	−0.09810	−4.187	0.27234	0.19491
All populations	98	65	104	0.985 ± 0.005	0.05101 ± 0.00168	20.147	−0.00311	−22.794	−0.54873	−0.38190

*N*—number of sequences analyzed; *H*—number of haplotypes; *S*—number of segregating sites; Hd—haplotype diversity; *π*—nucleotide diversity; *K*—average number of nucleotide differences; *D*—Tajima's statistic [45]; Fs—Fu's statistic [47]; D* and F*—Fu and Li's statistics [46].  *P < 0.10 (for *D*, D* and F*). Significance was determined using coalescent simulations in DnaSP version 4.0 (Rozas et al. 2003) [38]. ^†^Sites with gaps were completely excluded.

**Table 7:** Segregating sites (104 bp) in 395-bp segment of D-loop gene defining 65 haplotypes and their distribution across seven populations of *M. f. fascicularis*.

H aplotype												Locality						
	Nucleotide Positions											1	2	3	4	5	6	7
										1	1111							
	1	1111111112	2222222223	3333333334	4444444445	5555555556	6666666667	7777777778	8888888889	9999999990	0000							
	1234567890	1234567890	1234567890	1234567890	1234567890	1234567890	1234567890	1234567890	1234567890	1234567890	1234							
Hap_1	CCCCGACATA	GTTACTTGCG	GACCTAAGTT	CAATCATCTA	AAGTATCCCA	CCACGCCTAA	ACCCATACCA	ACCTGATCGT	CACACGGGGC	GCAGTACCTT	TACC	1						
Hap_2	..........	...G.....A	A.....G...	TG...G....	.G....T...	..........	..........	..........	..........	.T...G....	.C..	1						
Hap_3	..........	...G.....A	A.....G...	TG...G....	.G....T...	..........	..........	..........	..........	.T...G....	.C..	1						
Hap_4	...T.T....	A.........	A.....GACC	.....G....	GGA.......	....A.....	..........	..........	.....A....	.TG.......	.C..	1						
Hap_5	..........	..........	A.....G..C	.....G..C.	.......T..	....A.....	..T.......	..........	..........	.TG..G....	CC..	1						
Hap_6	..........	..........	A..T......	.........T	..........	....A.....	..........	..........	..........	..........	.C..	1						
Hap_7	..........	........T.	A.....G..C	T....G....	..A....T..	..........	..........	..........	..........	.T...G....	.C..			2				
Hap_8	..........	......C...	A........C	TG...G....	.G...CT...	.......C..	..........	..........	..........	.T...G....	.C..		1					
Hap_9	..........	..........	A........C	TG...G....	.G...CT...	.......C..	..........	..........	..........	.T...G....	.C..		3					
Hap_10	..........	..........	A........C	TG...G....	.G...CT...	.......C..	..........	..........	..........	.T...G....	.C..	1						
Hap_11	T.........	.......A..	..........	..........	..........	..........	..........	..........	..........	.....G....	....	1						
Hap_12	....A.....	..........	A........C	TG........	.G...CT...	....A..C..	..........	..........	..........	.T...G....	.C.T	1						
Hap_13	..........	..........	A.....G..C	TG...G....	.G........	.......C..	.....C....	..........	..........	.TG..G....	.C..	1						
Hap_14	..........	.........A	.G.T......	T.........	....GC....	....A...G.	....G.....	..........	..........	.TG..G....	.C..	2						
Hap_15	...T......	..C.......	A.........	T.........	....G.....	........G.	....G.....	..........	..........	.T...G....	.C..	1						
Hap_16	..........	.........A	......G...	T.........	....G.....	........G.	....G.....	..........	..........	.T...G....	.C..	1						
Hap_17	........C.	..C.......	A.....G..C	T.........	....G.....	........G.	G...G.....	..........	..........	.T...G....	.C..	1						
Hap_18	..........	.........A	A.....G...	T.........	....G.....	........G.	....G.....	..........	..........	.T...G....	.C..	1						
Hap_19	..........	.........A	A.........	T.........	....G.....	........G.	....G.....	..........	..........	.T...G....	.C..	1						
Hap_20	..........	..........	A.....G..C	T.........	.......T..	..........	.......T..	..........	..........	.....G....	.C..			4				
Hap_21	..........	..........	A.....G..C	T....G....	.......TT.	..........	..........	..........	.....A....	.T...G....	.C..			1				
Hap_22	...T......	..........	A.....G..C	T.........	.......T..	..........	.......T..	..........	..........	.....G....	.C..			1				
Hap_23	.T.....T..	..C.......	A..T..G..C	....T.....	..A....T..	TTCT.TT...	.....C...G	...C......	..TT......	.TG...T...	.C..				1	1		
Hap_24	.T....TT..	A.C......A	A..T..G...	....T.....	..A....TT.	T.CT.TT...	.....C...G	G..C..C...	..TT......	.TG...T...	.C..				2			
Hap_25	.T....TT..	A.C......A	A..T..G...	....T.C...	..A....TT.	T.CT.TT...	.....C...G	G..C..C...	..TT......	.TG...T...	.C..				1			
Hap_26	.T.....TC.	..........	A.....G...	....T..TC.	G.A....T.G	T..T.T....	.....C...G	...C......	..T.......	.TG...T...	.C..				1			
Hap_27	.T.....T..	..C.......	A..T..G..C	....T.....	G.A....T..	TTCT.TT...	.....C...G	...C......	..TT......	.TG...T...	.C..				1	1		
Hap_28	.T.....T..	..C.......	A..T..G..C	....T.....	..A....T..	TTCT.T....	.....C...G	...C......	..TT......	.TG...T...	.C..				1			
Hap_29	.T.....T..	..C...C...	A..T..G..C	....T.....	G.A....T..	TTCT.TT...	.....C...G	...C......	..TT......	.TG...T...	.C..				1	1		
Hap_30	.T....TT..	A.C......A	A..T..G...	....T.....	..A....TT.	T.CT.TT...	.....C....	G..C..C...	..TT......	.TG...T...	.C..				1			
Hap_31	.T.....T..	..C.......	A..T..G..C	....T.....	..A....T..	TTCT.TT..G	.....C...G	...C......	..TT......	.TG...T...	.C..				1			
Hap_32	.T.....T.G	.........A	A.....G...	....T..TC.	G.A....T.G	T..T.T....	.....C...G	...C......	..T.......	.TG...T...	.C..				1			
Hap_33	.T.....T..	..C.......	A..T..G..C	....T.....	..A.......	TTCT.TT...	.....C...G	...C......	..TT......	.TG...T...	.C..				1	1		
Hap_34	.T...T.T..	..C.......	A.....G..C	....T.....	..A....T..	TTCT.TT...	.....C...G	...C......	..TT......	.TG...T...	.C..				1			
Hap_35	.T....TT..	A.C......A	A..T......	....T.....	..A....TT.	T.CT.TT...	.....C....	G..C..C...	..TT......	.TG...T...	.C..				1			
Hap_36	.TT....T..	..C.......	A..T..G..C	....T.....	..A....T..	TTCT.T....	.....C...G	...C......	..TT......	.TG...T...	.C..				1			
Hap_37	.T....TTC.	.........A	A..T..GAC.	....T..TC.	G.A....T.G	T..T.T.C..	.....C...G	...C......	..T.....A.	ATG...T...	.C.T					2		
Hap_38	.T.....T..	..C......A	..TT.TG...	..G.T.....	..A....TT.	..CT.TT...	.....C....	...C......	..TT......	ATG...T...	.C..					1		
Hap_39	.T....TT..	A.C......A	A..T..G...	....T.....	..A....TT.	T.CT.TT...	.....C...G	G..C..C...	..TT......	.TG...T..C	.C..					1		
Hap_40	.T.....T..	..C......A	...T.TG...	..G.T.....	..A....TT.	..CT.TT...	.....C....	...C......	..TT......	.TG...T...	.C..					1		
Hap_41	.T....TTC.	.........A	A..T..GAC.	....T..TC.	G.A....T.G	T..T.T.C..	.....C...G	G..C......	..T.....A.	ATG...T...	.C.T					1		
Hap_42	.T.....T..	..C.......	A..T..G..C	....T.....	..A..C.T..	TTCT.TT...	.....C...G	...C......	..TT......	.TG...T...	.C..					1		
Hap_43	.T....TT..	A.C......A	A..TC.G...	....TG....	..A....TT.	T.CT.TT...	.....C...G	G..C..C...	..TT......	.TG...T...	.C..					1		
Hap_44	.T.....T..	..C.......	A..T.TG..C	..G.T.....	..A....TT.	T.C..TT...	.T...C....	...C......	..TT......	.TG...T...	.C..					1		
Hap_45	.T.....T..	..C..C....	A..T..G..C	....T.....	G.A....T..	TTCT.TT...	.....C...G	...C......	..TT...A..	.TG...T...	.C..					1		
Hap_46	.T.....T..	..C.......	A..T..G..C	....T.....	..A.......	TTCT.TT...	.....C...G	...C....A.	..TT......	.TG...T...	.C..					1		
Hap_47	.T.....TC.	.........A	A.....G...	....T..TC.	G.A....T.G	T.GT.T....	.....C...G	...C......	..T.......	.TG...T...	.C..						5	
Hap_48	.T....TT..	AC..TC...A	A..T..G...	....T...C.	..A....TT.	T.CTAT...G	.....C..TG	...C......	..T.......	.T....T...	.C..							8
Hap_49	.T....TT..	AC..TC...A	A..T..G...	....T...C.	..A....TT.	T.CTAT...G	.....CT.TG	...C......	..T.......	.T....T...	.C..							1
Hap_50	.T....TT..	A...TC...A	A..T..G...	....T...C.	..A....TT.	T.CTAT...G	.....C..TG	...C......	..T.......	.T....T...	.C..							1
Hap_51	.T....TT..	A...TC...A	A..T..G...	....T...CG	..A....TT.	T.CTAT...G	.....C..TG	...C......	..T.......	.T....T...	.C..							2
Hap_52	.T....TT..	A...TC...A	A..T..G...	....T...C.	..AC......	T.CTAT.C..	.....C..T.	...C......	..T.GA...G	.T....T.G.	.CA.							1
Hap_53	.T....TT..	A...TC...A	A..T..G...	....T...C.	..AC......	T.CTAT.C..	.....C..T.	...C......	..T.......	.T..G.T.G.	.CA.							1
Hap_54	.T....TT..	A...TC...A	A..T..G...	....T...C.	..AC......	T.CTAT.C..	.....C..T.	...C......	..T.......	.T....T.G.	.CA.							1
Hap_55	.T....TTC.	A...TC...A	A..T..G...	....T...C.	..A....TT.	T.CT.T...G	.....C..TG	...C......	..T.......	.T....T...	.C..							3
Hap_56	.T....TT..	.........A	A..T..G...	....T.C.C.	..A....TT.	T.CTAT...G	.....C..TG	...C......	..T.......	.T....T...	.C..							5
Hap_57	.T....TT..	.........A	A..T..G...	....T.C.C.	..A....TT.	T.CTAT...T	.....C..TG	...CA...AC	TTT..AA...	.T....T...	.C..							1
Hap_58	.T....TT..	.........A	A..T..G...	....T.C.C.	..A....TT.	T.CTAT....	.....C..TG	.TTCAC.TAC	T.T...A...	.T.T..TT..	.C..							1
Hap_59	.T....TT..	AC..TC...A	A..T..G...	...CT...C.	..A..C.TT.	T.C.A.....	...T.C..TG	...C......	..T.......	.T....T...	.C.T							1
Hap_60	.T.....TC.	A...TC....	A.....G...	....T.C.C.	..A....TT.	T.CTAT...G	.....C..TG	...C......	..T.......	.T....T...	.C..							1
Hap_61	.T.....TC.	A...TC...A	A.....G...	....T.C.C.	..A....TT.	T.CTAT...G	.....C..TG	...C......	..T.......	.T....T...	.C..							1
Hap_62	.T.....T..	A..GTC...A	A.....G...	....T.C.C.	..A....TT.	T.CTAT...G	.....C..TG	...C......	..T.......	.T....T...	.C..							1
Hap_63	.T....TT..	A...T....A	A..T..G...	...CT...C.	..A....TT.	..CTAT...G	.....C.TT.	...C......	..T.......	.T....T...	.C..							2
Hap_64	.T.....TC.	A...TC....	A..T..G...	....T.C.C.	..A....TT.	..CTAT...G	.....C..TG	...C......	..T.......	.T....T...	.C..							1
Hap_65	.T.....TC.	A....C....	A..T..G...	....T.C.C.	..A....TT.	..CTAT...G	.....C..TG	...C......	..T.......	.T....T...	.C..							1

∗Locality information: 1—Indonesia, 2—Mauritius, 3—Philippines, 4—Cambodia, 5—Vietnam, 6—Thailand, 7—Malaysia.
